# New Pacing Techniques and Non-Invasive Methods That May Improve Response to Cardiac Resynchronization Therapy

**DOI:** 10.3390/jcdd11070208

**Published:** 2024-07-03

**Authors:** András Vereckei

**Affiliations:** Department of Medicine and Hematology, Semmelweis University, Szentkirályi u. 46, 1088 Budapest, Hungary; vereckei.andras@med.semmelweis-univ.hu; Tel.: +36-1-266-0926

Although cardiac resynchronization therapy (CRT) is an evidence-based effective therapy of symptomatic heart failure with reduced ejection fraction (HFrEF), refractory to optimal medical treatment and associated with intraventricular conduction disturbance, the non-response rate to CRT is still around 30%. In patients with HFrEF, intraventricular conduction disturbance results in electrical ventricular dyssynchrony, which further deteriorates systolic ventricular function. The main determinant of CRT outcome is the presence or absence of significant ventricular electrical dyssynchrony (which results in ventricular mechanical dyssynchrony) and the ability of the applied CRT technique to eliminate it [1,2]. CRT is not effective in mechanical ventricular dyssynchrony without electrical dyssynchrony (e.g., that occurs in the presence of myocardial scar or myocarditis); it can only eliminate mechanical dyssynchrony due to underlying primary electrical ventricular dyssynchrony [3]. Therefore, theoretically, electrocardiological and electrophysiological criteria are probably more important in patient selection for CRT than imaging criteria. The current guidelines [4] recommending only QRS duration and morphology for patient selection for CRT and not recommending imaging modality criteria after the disappointing results of the PROSPECT trial [5], investigating the role of echocardiographic mechanical dyssynchrony criteria for patient selection for CRT, comply with this principle. However, there may be two main reasons for the still non-negligible non-response rate to CRT. The first reason is that QRS duration and morphology are not optimal criteria to assess the presence or absence of ventricular electrical dyssynchrony. The second reason is that the current biventricular pacing CRT (BIVP-CRT) technique, using apical right ventricular (RV) and epicardial left ventricular (LV) pacing in the posterolateral or lateral area, although significantly improving ventricular dyssynchrony and resulting in a more synchronious ventricular activation, cannot completely restore the physiological ventricular activation and synchrony [6]. Another problematic property of the current BIVP-CRT technique is that it was originally devised to eliminate dyssynchrony caused by left bundle branch block (LBBB) pattern. It is not appropriate in patients with pure, typical right bundle branch block (RBBB) pattern, and may not be appropriate in some patients with nonspecific intraventricular conduction disturbance (NICD) pattern, for eliminating dyssynchrony. This is because the latest activated LV site in patient with pure, typical RBBB and in some patients with NICD patterns is far away from the standard LV pacing site used in the BIVP-CRT technique [2]. Therefore, we can decrease the non-response rate to CRT if we can apply better pre-CRT patient selection criteria than QRS duration and morphology, that can more accurately assess the presence or absence of significant ventricular electrical dyssynchrony. The other possible way to decrease the non-response rate to CRT is the application of novel CRT techniques, such as conduction system pacing (CSP) [His bundle pacing (HBP) and left bundle branch area pacing (LBBAP)]. These, in contrast to BIVP-CRT technique, can completely restore physiological ventricular activation [6]. Another possibility to reduce the number of non-responders to CRT is to apply an individualized BIVP-CRT technique, when the LV pacing electrode is positioned at the site or very close to the site of the latest activated LV region [7]. Another advantage of CSP compared with BIVP-CRT technique is that the latter has proarrhythmic potential due to non-physiologic ventricular activation. In contrast to that, CSP potentially abolishes this proarrhythmic effect by restoring physiological ventricular activation [6]. This Special Issue, under the title “New pacing techniques and non-invasive methods that may improve response and patient selection to cardiac resynchronization therapy”, discusses novel non-invasive techniques and methods that can better assess the presence of ventricular electrical dyssynchrony than QRS duration and morphology and provides a review of CSP.

Simon A et al., (contribution 1) discuss novel 12-lead ECG signs that may improve the prediction of response to CRT, such as the presence of masquerading bundle branch block, the higher R wave amplitude and R/S ratio in lead V_6_ [8], increased intrinsicoid deflection (ID) in lateral leads [9], the novel intraventricular [(aVLID-aVFID)/QRS duration] and interventricular [(V_5_ID − V_1_ID)/QRS duration] ECG dyssynchrony criteria > 25% [10], and a QR_max_ index ≥ 120 ms, that is the longest QR index in the limb leads, which is the interval from the onset of the QRS to the R wave offset, defined as the intersection between the descending limb of the R wave and the baseline [11]. They also delineate that a Q-LV interval > 95 ms, measured during CRT from the onset of the QRS on the 12-lead ECG to the first large peak of the LV electrogram obtained from the LV pacing lead, predicts response to CRT as well [12]. An RV-LV interval ≥ 70 ms, determined during unpaced beats during CRT by measuring the interval between the first large positive or negative peaks of the RV and LV electrograms, also predicts response to CRT [13].

Eerenberg F et al., (contribution 2) discuss the potential use of the vectorcardiographic QRS area in pre-CRT patient selection and in LV lead implantation guidance during CRT. A QRS area > 109 μVs predicts a response to CRT, and a greater reduction in QRS area during CRT indicates a more optimal position of the LV electrode [14].

Abu-Alrub S et al., (contribution 3) summarize the role of electrocardiographic imaging, a combination of body surface mapping with computed tomography and a special software, which corresponds to a non-invasive epicardial electrophysiological examination, in patient selection for CRT and the optimization of LV lead placement, and the possible use of ECG belt, an innovative body surface mapping system, in patient selection for CRT.

Nguyen UC et al., (contribution 4) outline the clinical application of ultra-high-frequency ECG (UHF-ECG) in dyssynchrony assessment for patient selection to CRT and during cardiac pacing to show which pacing modality preserves best ventricular synchrony. UHF-ECG displays the ventricular activation sequence analyzing the ultra-high-frequency (150–1000 Hz) components of ventricular myocyte action potentials’depolarization phase (phase 0) using either the standard chest leads (V_1_–V_6_) or 8 chest leads (V_1_–V_8_).

The above-mentioned promising methods—vectorcardiographic QRS area, electrocardiographic imaging, ECG belt and UHF-ECG—can decrease non-response to CRT by providing a more accurate assessment of ventricular electrical dyssynchrony than QRS duration and morphology.

Dutta et al., (contribution 5) describe the most promising echocardiographic mechanical dyssynchrony parameters that can be applied after the disappointing PROSPECT trial for better patient selection for CRT, mostly in patients with non-LBBB pattern, who respond less well to CRT, but also in patients with LBBB pattern. These potentially valuable echocardiographic dyssynchrony parameters that can identify CRT responders are as follows: (1) the mechanical dispersion index (standard deviation of the averaged time to peak longitudinal strain) ≥ 60 ms, (2) the time difference in peak septal wall to posterior wall radial strain ≥ 130 ms, (3) the presence of apical rocking, (4) the presence of septal flash, and (5) the classical pattern of mechanical discoordination in patients with LBBB pattern (early contraction of septal wall and simultaneous passive stretching of the opposite lateral wall, which contracts late, achieving its peak contraction after aortic valve closure).The estimation of myocardial work, which is a more sensitive, afterload-independent parameter of LV systolic function and ventricular dyssynchrony than the afterload-dependent ejection fraction and global longitudinal strain, is also a promising parameter to predict response to CRT [15,16]. The results of the Markers and Response to CRT (MARC) study [17] support that one previously mentioned echocardiographic parameter and the vectorcardiographic QRS area are potentially valuable in the identification of CRT responders, because they found that independent predictors of CRT response [defined as a ≥15% reduction of LV end systolic volume (LVESV)] were as follows: younger age (<60 years); larger QRS area; longer interventricular mechanical delay (>40 ms); calculated as the difference between LV and RV pre-ejection intervals; measured from the QRS complex to the onset of aortic and pulmonic flow; and the presence of apical rocking.

Cano O et al., (contribution 6) and Castagno D et al., (contribution 7) discuss CSP (HBP and LBBAP) in detail, which is gradually becoming a potential real alternative to the standard BIVP-CRT technique due to its ability to restore the physiologic ventricular activation.
